# tsRNAs: A Prospective, Effective Therapeutic Intervention for Neurodegenerative Diseases

**DOI:** 10.1111/cns.70177

**Published:** 2024-12-17

**Authors:** Tianqi Li, Hui Zhen, Weiwei Wu, Fengtang Yang, Zhonghong Cao

**Affiliations:** ^1^ School of Life Science and Medicine Shandong University of Technology Zibo Shandong China

**Keywords:** neurodegenerative diseases, nucleic acid drug, regeneration, tsRNAs

## Abstract

**Background:**

Neurological disorders known as neurodegenerative diseases (NDDs) result in the slow loss of neurons in the central nervous system (CNS) or peripheral nervous system (PNS), as well as the collapse of neural networks in terms of structure and function. NDDs are expected to surpass cancer as the second biggest cause of mortality by 2040, according to World Health Organization (WHO) estimations. Neurons cannot effectively regenerate themselves because they are terminally differentiated. Accordingly, it is challenging to find medications that could stop or slow neurodegeneration.

**Main body:**

The tsRNAs are a type of small non‐coding RNAs derived from mature tRNAs or tRNA precursors. tsRNAs control gene expression and have a role in many physiological and pathological processes, including neurological illnesses. Antisense oligonucleotides are effective therapeutic agents for neurological diseases, and they may be the treatment of choice for neurodegenerative diseases in the future. Here, we review the biogenesis of tsRNA, its physiological and pathological functions in the central nervous system and neurological disorders, and its prospective use as a nucleic acid medication to treat NDDs, providing theoretical support and guidance for further exploration of tsRNAs in therapeutic intervention.

**Conclusion:**

tsRNAs are emerging as important regulatory molecules in neurodegenerative diseases. Understanding the functions of tsRNAs in neurodegenerative diseases may provide new insights into disease mechanisms and lead to the development of novel treatment strategies.

## Graphical abstract

Transfer RNA‐derived small RNAs (tsRNAs) are generated through the cleavage of transfer RNAs. tsRNAs modulate gene expression through transcriptional and translational mechanisms. tsRNAs are implicated in the pathogenesis of neurodegenerative disorders. tsRNAs have potential applications as nucleic acid therapeutics for the treatment of neurodegenerative diseases.

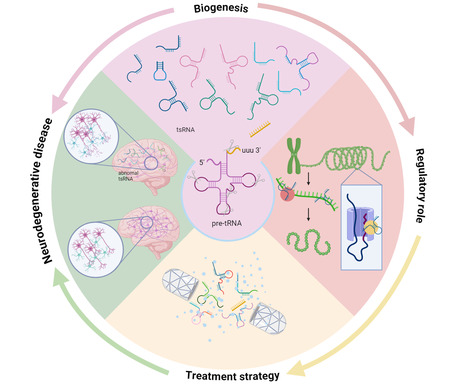

**Abbreviations**
AD: Alzheimer's disease
AGO: Argonaute
AGOs: Argonaute family proteins
ALS: amyotrophic lateral sclerosis
Ang: angiopoietin
ASOs: antisense oligonucleotides
BSTSF: Bushen Tiansui formula
BYHWD: Buyang Huanwu decoction
CNS: central nervous system
Cyt C: cytochrome C
Cyt C‐RNP: Cyt C‐ribonucleoprotein complex
ERVs: endogenous retroviruses
G4: G tetramer
HD: Huntington's disease
HEK: human embryonic kidney
LATS2: large tumor suppressor kinase 2
NDDs: neurodegenerative diseases
nt: nucleotides
PBS: primer‐binding sequence
PD: Parkinson's disease
PNS: peripheral nervous system
pre‐mRNA: precursor mRNA
reaEGCs: reactive ependyma glial cells
RG4: RNA G‐quadruplex
SGs: stress granules
SHOT‐tRNA: sex hormone‐dependent tRNA
siRNAs: small interfering RNAs
tiRNA: tRNA‐derived stress‐induced RNAs
TSEN: tRNA splicing endonuclease
tsRN: tRNA‐derived small RNA
tsRNAs: tRNA‐derived small RNAs
WHO: World Health Organization
XFZD: Xuefu zhuyu decoction
YB‐1: Y‐box binding protein 1

## 1 Introduction

Neurological disorders known as neurodegenerative diseases (NDDs) result in a progressive loss of neuronal structure and function, which impairs cognition and causes dementia. Numerous illnesses, including age‐related Parkinson's disease (PD) and Alzheimer's disease (AD), genetically‐related Huntington's disease (HD), and amyotrophic lateral sclerosis (ALS) with no known cause, can lead to partial or total degeneration of the nerves. There are a few essential characteristics in these disease states: (1) the misfolding and aggregation of protein that results in neurofibrillary tangles, neuritic plaques, and cytotoxicity; (2) the proliferation and activation of microglial cells and neuroinflammation; (3) damage to neurons, disturbance of synaptic connections, and compromised signal transduction processes; (4) mitochondrial malfunction, oxidative stress, and autophagy defects.

Being the most complex organ in the body, injury to the nervous system can have a major effect on an individual's health. Stem cells found in the central nervous system of mice can specialize and produce new neurons to heal injured central nerves [1, 2, 3, 4]. Salamander reactive ependymoglial cells (reaEGCs) play a regulatory role in the regeneration of injured neurons. During telencephalic regeneration in salamanders, reaEGCs are able to proliferate at the wound site and then differentiate into mature neurons to rebuild the injured brain tissue [5]. It is generally accepted that mature brain cells have a limited ability for regeneration and that neurons cannot efficiently regenerate themselves. Thus, the investigation of brain stem cell differentiation and proliferation will affect the treatment of neurodegenerative illnesses in the future.

tRNA‐derived small RNAs (tsRNAs) is a type of short, non‐coding small RNAs that have been found to be highly conserved throughout evolution. These molecules are produced when enzymes like Angiogenin (ANG), Dicer, RNase Z, and RNase P cleave mature tRNAs or tRNA precursors [6, 7, 8]. Before, tsRNA was thought to be an arbitrary, degraded tRNA fragment with no function. Recent advances in high‐throughput sequencing technology have revealed the tsRNA expression pattern of both physiological and pathological conditions. Notably, these profiles are highly responsive to environmental stressors [9, 10]. tsRNAs are essential regulators in pathological and physiological settings, including neurogenesis and neurodegenerative illnesses [11].

In this review, we provide an overview of the biogenesis and regulatory roles of tsRNAs, as well as its physiological and pathological roles in neurological disorders. We also discuss the potential use of tsRNA nucleic acids in treating neurodegenerative diseases and neuronal regeneration following nerve damage, supplying theoretical backing and direction to continue exploring novel therapeutic avenues for neurodegenerative illnesses.

## 2 Biogenesis of tsRNAs

**FIGURE 1:**
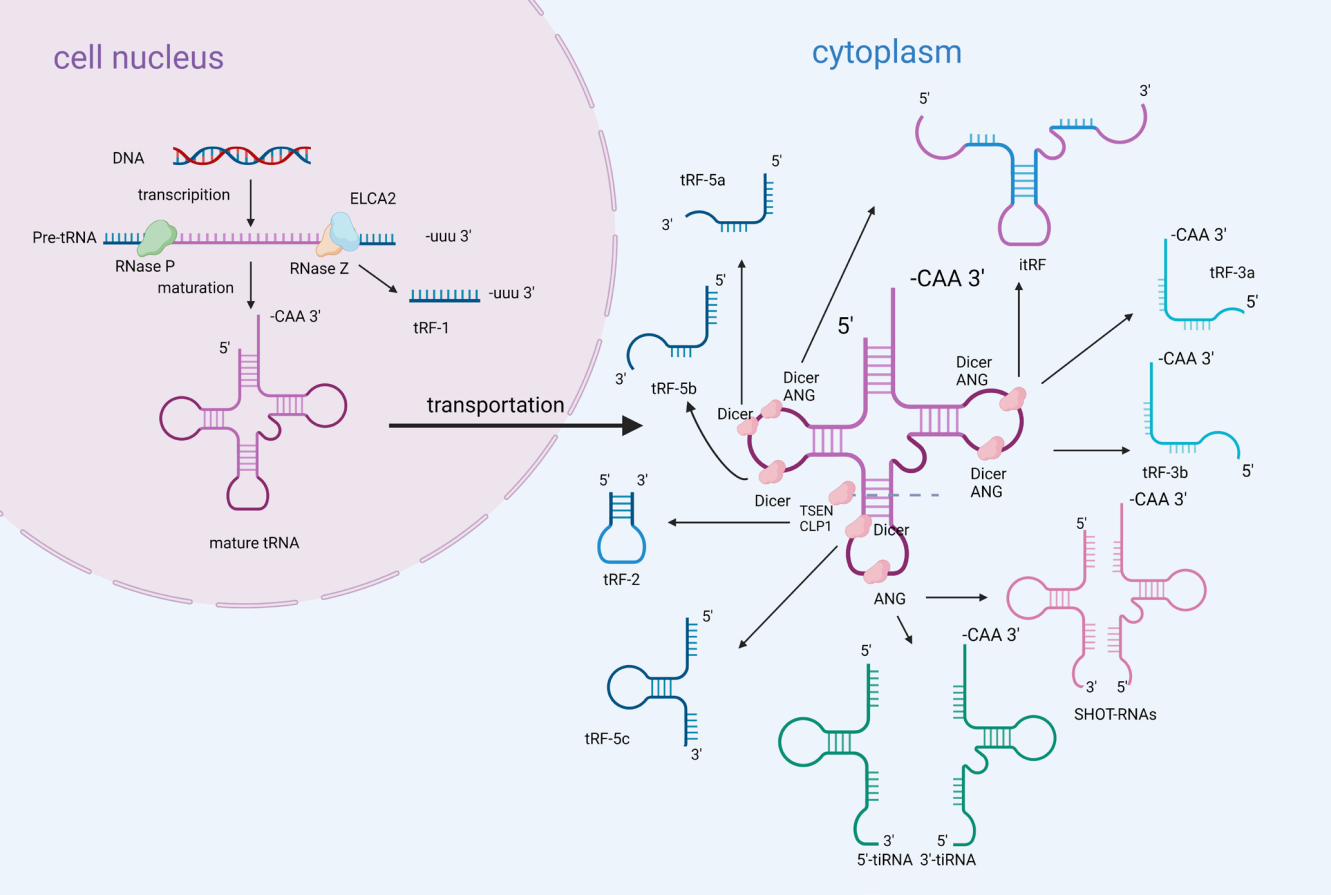
The process of tsRNA generation. The tRNA precursor formed by nuclear transcription is excised from the 5′ end of the leading sequence by RNase P, and from the 3′ end of the trailing sequence by RNase Z and ELAC2, generating tRF‐1. Nucleotidyl transferases add CCA bases to the 3′ end of tRNA, generating the mature tRNAs. Dicer, ANG, TSEN, CLP1, and other enzymes further cleave the different positions of mature tRNA to generate different types of tsRNA, such as tRF‐2, tRF‐3, tRF‐5, itRF, 3′ tiRNA, 5′ tiRNA, SHOT‐RNA and so on.

tsRNAs is a family of evolutionarily highly conserved non‐coding small RNAs derived from tRNA, also known as tRFs/tRNA‐derived fragments [12, 13]. As shown in Figure 1, tRNA precursors are produced by RNA polymerase III from the tRNA gene [14]. The mature tRNA is created by employing RNase P to remove the leader sequence from the 5′ end of the tRNA precursor, RNase Z to remove the trailer sequence from the 3′ end, and tRNA nucleotide transferase to add the CCA base at the 3′ end. tsRNAs are created by cleaving tRNA precursors or Mature tRNA using Angiogenin, Dicer, RNase Z, and RNase P, etc. [6, 7, 8]. The tsRNAs are created by shearing off the 3′ and 5′ ends of the tRNA precursor sequence. Mature tRNAs are transported into the cytoplasm by exonuclear transport proteins [15]. tRNAs can be roughly classified into two groups: those derived from mature tRNAs and those derived from tRNA precursors (Table 1).

**TABLE 1:** Types of tsRNAs.

Name	Cutting position	Enzymes	References
tRF‐1	3′ tailer	RNase Z, ELAC2	[13, 16]
tRF‐2	Anticodon loop	Dicer	[17]
tRF‐3a	T loop	Dicer	[18]
tRF‐3b	T loop	Dicer	[18]
tRF‐5a	D loop	Dicer	[18]
tRF‐5b	D loop	Dicer	[18]
tRF‐5c	D loop	Dicer	[18]
i‐tRF	D loop, T loop	Dicer, ANG	[7]
5′ tiRNA	Anticodon loop	ANG	[7]
3′ tiRNA	Anticodon loop	ANG	[7]
5′ SHOT‐RNA	Anticodon loop	ANG	[19]
3′ SHOT‐RNA	Anticodon loop	ANG	[19]

### 2.1 tsRNAs From tRNA Precursor

RNase Z and ELAC2 cleave the precursor tRNA's 3′‐UUU end to generate tRF‐1 during the maturation process of the precursor tRNA. Of these, tRF‐1001 is the first recognized 3′u tRF derived from the tRNA precursor of SerTGA [13]. tRF‐1 expression is essential for cell viability and is controlled by cell proliferation [13]. While tRF‐1 is expressed less in normal tissues, it is highly expressed in a variety of cancer tissues [20, 21, 22]. This difference suggests that a certain type of tRF‐1 is selectively processed or retained in tissues for specific biological functions [13]. It has been demonstrated that tRNA‐1 is generated in the nucleus and then transported to the cytoplasm, but the mechanism of its nuclear export is not clear [16].

### 2.2 tsRNAs From Mature tRNA

There are two primary classes of tsRNAs (5′ tsRNA and 3′ tsRNA) produced from mature tRNAs. The former contains the mature tRNA 5′ leader, while the latter contains the mature tRNA 3′ tailer [23]. 5′ tsRNA consists of tRF‐5 and 5′ tRNA‐derived stress‐induced RNAs (tiRNA), and 3′ tsRNA, tRF‐3 and 3′ tiRNA.

Dicer enzyme cleavage at the D‐loop or at the rod position between the D‐loop and the anticodon loop results in the tRNA fragment known as tRF‐5, which is 14–30 nucleotides (nt) in length. Depending on the cutting position, they can be classified into three categories: tRF‐5a, tRF‐5b, and tRF‐5c [18].

tRF‐3 contains 18–22 nt. Their 5′ end is in the T‐loop and their 3′ end matches the 3′ end of the mature tRNA, and both of their ends terminate in CCA. It is produced by cleavage of the T‐loop of mature tRNA by ANG, Dicer, or members of the ribonuclease family [24].

tiRNA is formed by the cleavage of a related molecule at the anticodon loop of the mature tRNA. Two components are produced as a result of the cleavage: 5′ tiRNA and 3′ tiRNA; they are also called 5′ tRNA half and 3′ tRNA half, respectively. In mammals [7], the process is completed by ANG cleavage, while in yeast, the process is carried out by the nucleic acid endonuclease Rny1p [25]. Studies have shown that tiRNA is mainly produced under stress conditions such as starvation, nutritional deficiencies, oxidative stress, heat shock, and UV exposure [7, 26, 27].

In addition, there is a type of tiRNA with sex hormone‐dependent properties, called sex hormone‐dependent tRNA (SHOT‐tRNA), which includes 5′ SHOT‐tRNA and 3′ SHOT‐tRNA. SHOT tRNA is a special type of tiRNA produced by ANG. A phosphate is present at the 5′ end of the 5′ SHOT‐tRNA, while a 2′,3′‐cyclic phosphate is present at the 3′ end. A Hydroxyl Group is present at the 5′ end of the 3′ SHOT‐tRNA, and an amino acid is present at the 3′ end. They typically aggregate in high quantities in breast cancer cells. It has been demonstrated that SHOT‐RNA levels often drop upon depletion of the sex hormone receptors, ERα and AR. Accumulation of SHOT‐RNA promotes cell proliferation [19].

Additionally, several tRNA derivatives, including tRF‐2 and i‐tRF, have been discovered although their route of synthesis is unknown. tRF‐2 contains anticodon loops and stem sections [28]. It has been demonstrated that tRF‐2 is induced by hypoxic conditions. They can bind to YBX28 and segregate it from oncogenic transcripts, thus acting as an oncostatic agent [17, 28]. The i‐tRF includes anticodon loops and variable loops of mature tRNA. It has been demonstrated that i‐tRF expresses differently in normal and tumor cells, indicating that it may be associated with tumorigenesis [29].

## 3 Regulatory Role of tsRNAs

The tsRNAs carry out their biological functions using multiple approaches, including translation regulation, cell cycle modulation, and regulation of chromatin and epigenetic modification.

### 3.1 Regulating Translation

#### 3.1.1 Binding to Argonaute

**FIGURE 2:**
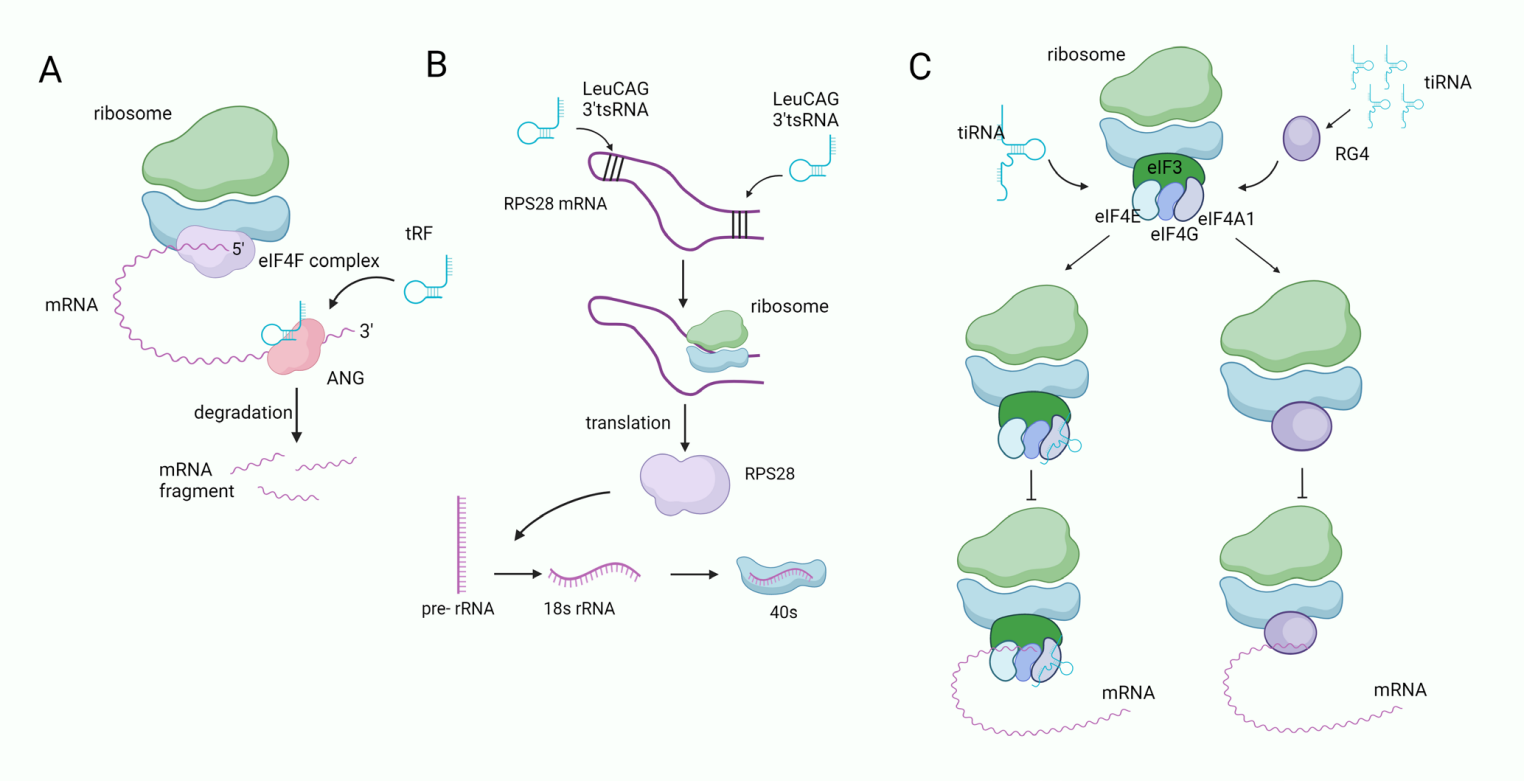
tsRNA regulates the process of translation. (A) tRF enters the ANG complex. By interacting with the 3′ UTR of the mRNA to degrade the target mRNA, thus silencing the gene. (B) LeuCAG 3′ tsRNA unfolds the secondary structure of two targets of RPS28 mRNA. This enhances the translation of RPS28 mRNA, resulting in an increase in RPS28, which promotes the production of 18S rRNA to form more 40S ribosomal subunits. (C) The tiRNA monomer interacts with eIF4A1 in the eIF4F complex to reduce mRNA binding through the G‐quadruplex structure. tiRNAs form the RG4 complex between them through the TOG sequence at the 5′ end. RG4 is able to disrupt the scanning of the 40S ribosome for mRNAs by replacing the eIF4F complex.

Like miRNAs and siRNAs, tsRNAs can bind to Argonaute family proteins (AGOs) to mediate gene silencing [18, 30, 31]. tRF‐3003a binds to the AGO2/GW182 protein complex to produce an AGO RNA‐induced silencing complex (AGO‐RISC). AGO‐RISC then targets the 3′ UTR of JAK3 mRNA to silence the JAK3 gene [30]. In human embryonic kidney (HEK) 293 cells, it was shown that tRF‐5 and tRF‐3 are associated with Argonautes 1, 3, and 4. They target thousands of RNAs to silence genes by forming cross‐links with AGO proteins and associate with target RNAs via canonical seed sites [18]. tRF‐3 can enter the Ago complex to perform microRNA‐like gene silencing functions. It suppresses target gene expression by interacting with the 3′ untranslated region (3′ UTR) of target gene mRNAs (Figure 2A) [31]. The primary mechanism of tRF‐3‐mediated inhibition is the degradation of the target RNA, most likely by de‐adenylation/decapitation and exonuclease digestion of the proposed nucleic acid against the microRNA target. Argonaute proteins are more easily bound by tRF‐3 when miRNAs are [31]. In addition, dicer‐dependent tsRNAs direct AGO2 to specific target sites located in the intronic regions of nascent RNA, resulting in the down‐regulation of target genes [32, 33].

#### 3.1.2 Acting on Ribosomes

tsRNA is involved in the processing of ribosomal RNA (rRNA) to regulate translation. The ribosome, also known as the “protein‐producing machine”, is widely recognized for its critical function in protein translation. LeuCAG 3′ tsRNA is able to indirectly regulate the process of ribosome synthesis by modulating the translation of the ribosomal protein RPS28. LeuCAG 3′ tsRNA unfolds the secondary structure of two targets, the coding and non‐coding 3′ UTR sequences of the RPS28 mRNA, which enhances the synthesis of RPS28. Increased RPS28 synthesis promotes the 18s rRNA production, providing more 18s RNA for 40s ribosome synthesis (Figure 2B) [34]. In addition, Kim et al. [35] also found that in addition to RPS28, LeuCAG 3′ tsRNA enhances translation of the ribosomal protein RPS15. In addition, *Haloferax volcanii* produces Val‐tRF during alkaline stress, which competes with mRNAs and binds to their surrounding ribosomal small subunits, impairing protein biosynthesis [36]. It has been shown that Val‐tRF could bind to the 70S ribosome and 30S subunit of *H. volcanii* , but was less successful in targeting the 50S subunit, and inhibits translation in vitro by interfering with peptide bond formation [37]. The above studies show that tsRNAs can regulate translation by acting directly or indirectly on ribosomes.

#### 3.1.3 Substituting Translation Initiation Factor eIF4F Complexes

Three proteins known as eukaryotic initiation factors, eIF4A, eIF4B, and eIF4F, are required for the translation initiation of natural mRNAs [38]. Wu et al. [39] found that the interaction between 5′‐tiRNA‐Gln and EIF4A1 reduced the associated mRNA binding via an intramolecular G‐quadruplex structure and this process partially inhibited translation and hepatocellular carcinoma progression. In addition, oligonucleotide TOG sequences at the 5′ ends of 5′ tiRNA‐Alk and 5′ tiRNA‐Cys can form RNA G‐quadruplex (RG4) to replace the translation initiation factor eIF4F complex, thereby disrupting the scanning of 40S ribosomes on mRNA (Figure 2C) [40, 41].

### 3.2 Regulating Apoptosis

**FIGURE 3:**
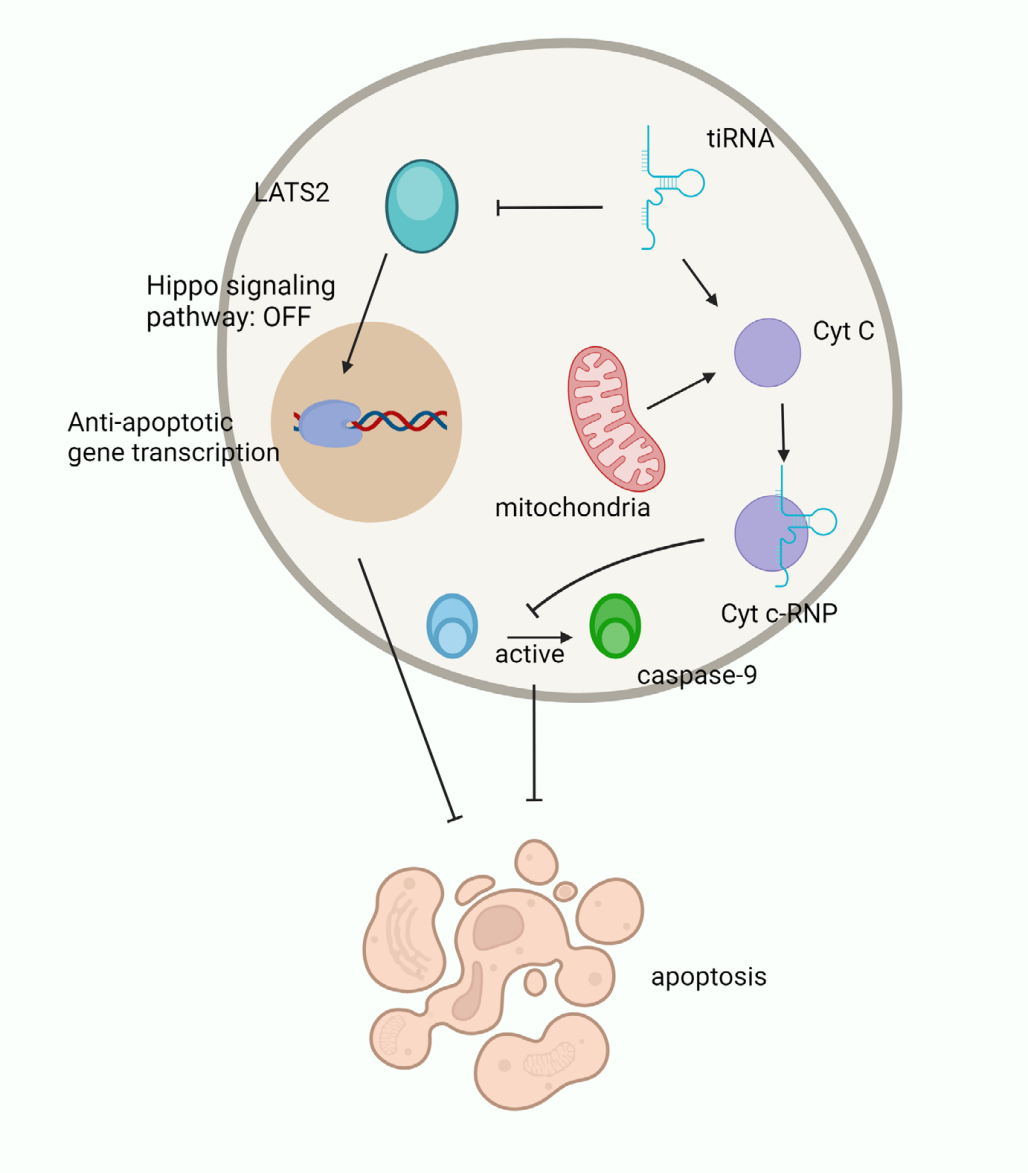
tsRNAs are involved in the process of apoptosis. tiRNA binds to Cyt C released from mitochondria to form the Cyt C‐RNA complex. This complex inhibits the activation of caspase‐9 and subsequently inhibits apoptosis. tiRNAs can also directly target LATS2 to inhibit the hippo signaling pathway, leading to an increase in the expression of anti‐apoptotic genes.

tsRNAs are able to regulate apoptosis by inhibiting apoptosis‐associated protein transport, binding to cytochrome C, or regulating the expression of related genes [42, 43, 44, 45, 46, 47]. To initiate apoptosis, cytochrome C (Cyt C) must be released from mitochondria and form apoptotic bodies [48]. Under hypertonic stress conditions, tiRNA from ANG cleavage can bind to nascent Cyt C specifically to form the Cyt C‐ribonucleoprotein complex (Cyt C‐RNP), which ultimately inhibits apoptosome formation [48]. In addition, it has been confirmed that tRNAPhe, tRNASer, and cytosolic tRNAGln inhibit caspase‐9 activation by binding to Cyt C directly and preventing apoptosis [43]. Tao et al. [45] reported that 5′ tiRNA‐His‐GTG was upregulated in human colorectal cancer tissues and positively correlated with tumor size. Additionally, it has been demonstrated that 5′ tiRNA‐His‐GTG specifically targets large tumor suppressor kinase 2 (LATS2) and suppresses the hippocampal signaling pathway to enhance the expression of genes that have anti‐apoptotic and pro‐proliferative properties (Figure 3).

### 3.3 Regulation of Chromatin and Epigenetic Modifications

**FIGURE 4:**
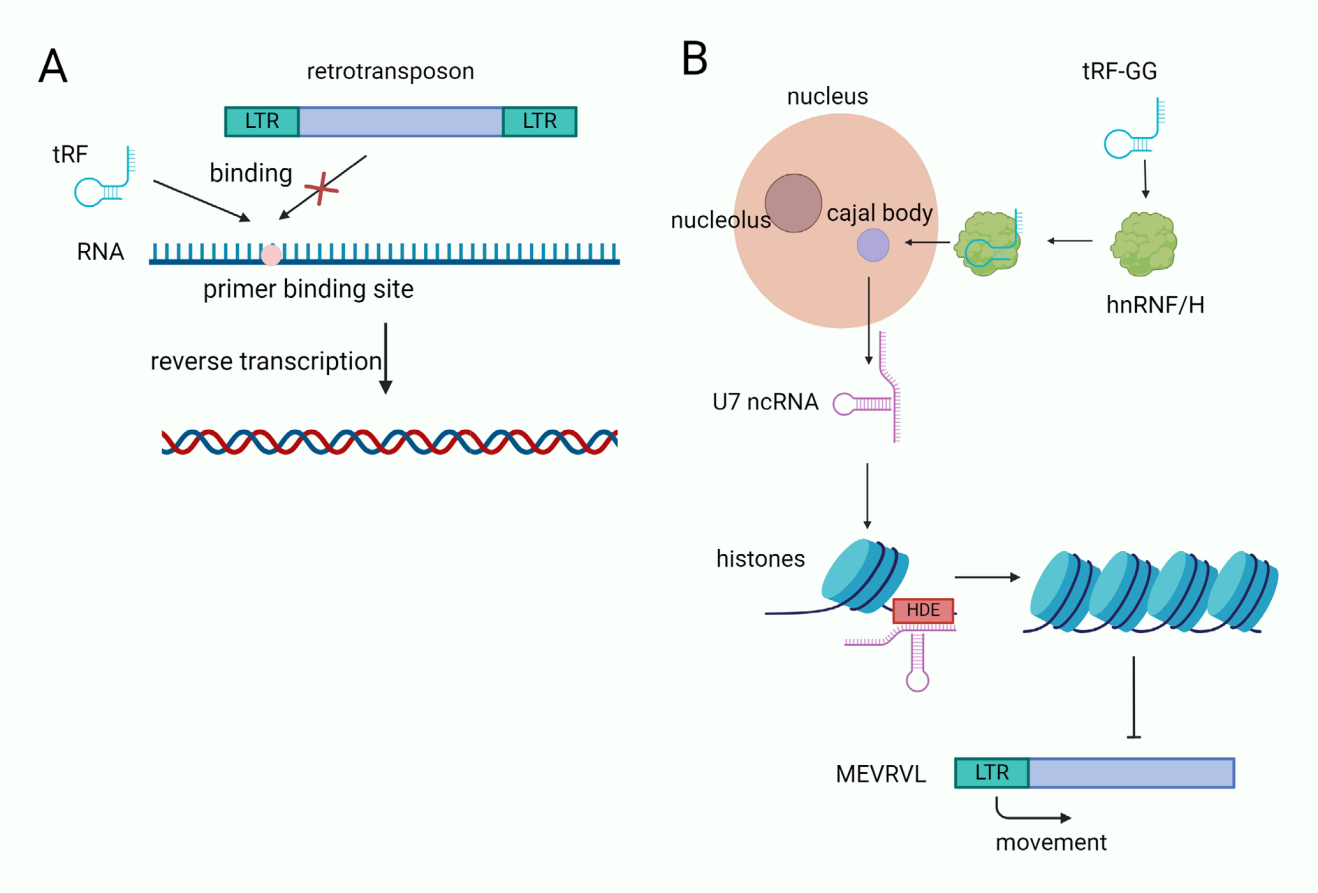
tsRNA regulates epigenetic processes. (A) tRF can bind to the primer binding site (PBS) of the retrotransposon to inhibit the binding of the retrotransposon to the PBS, thus inhibiting the reverse transcription process. (B) tRF‐GG binds to hnRNF/H and promotes the export of Cajal bodies to enhance the synthesis of U7 ncRNA. U7 ncRNA binds to histone downstream elements (HDE) to control histone processing. Increased histones inhibit the movement of endogenous reverse transcription elements (MEVRVL).

tsRNAs can influence transposable element activity or participate in intergenerational inheritance to participate in epigenetic processes [48, 49, 50]. During evolution, transposable elements have a significant impact on genetic diversity. It can move to different locations in the genome, leading to mutations [51]. Schorn et al. found that 18‐nt‐3′‐tRFs or 22‐nt‐3′‐tRFs (sequences complementary to endogenous retroviruses) inhibit their transposable activity. 18‐nt‐3′‐tRFs obstruct the reverse transcription process of viruses by competing with mature tRNAs for the primer‐binding sequence (PBS) of endogenous retroviruses (ERVs). 22‐nt‐3′‐tRFs inhibit the transcriptional activity of the ERVs by inducing an autonomous ERV protein‐coding mRNA that can cause post‐transcriptional silencing (Figure 4A) [50].

It has been demonstrated that sperm tsRNA is influenced by the paternal diet, which influences epigenetic inheritance [52, 53]. The tRF‐Gly‐GCC expression is elevated in spermatozoa during epididymal transport in mice treated with a low‐protein diet. The tRNA‐Gly‐GCC fragment inhibits more than 70 genes associated with the endogenous reverse transcription element (MERVL) in ES cells and embryos. This inhibition may have an impact on placental size or function, which could have an impact on metabolism later on as a result of placental changes [53]. Chen et al. [49] demonstrated that high‐fat diets contribute to the development of obesity, glucose intolerance, and insulin resistance and that 5′ tsRNA in spermatozoa can mediate the transfer of acquired metabolic disorders from the father to the offspring. Furthermore, tRF‐GG directly binds to hnRNF/H, the U7 RNA binding protein, to facilitate Cajal body export and guarantee adequate U7 synthesis. This allows sufficient histones to completely repress endogenous reverse transcription elements in the genome, affecting early embryo development (Figure 4B).

## 4 tsRNA Is a Promising Target for the Treatment of Neurodegenerative Diseases

Neurodegenerative diseases are characterized by the progressive loss of selectively susceptible neuronal populations. Based on their pathological features, they can be classified as amyloidosis, tauopathies, alpha synucleinopathies, and TDP‐43 proteinopathies [54]. Many studies have demonstrated that tsRNAs play an important role in the development of neurodegenerative diseases. The following section summarizes the pathological role of tsRNAs in neurodegenerative diseases and their promise for neurodegenerative disease treatment.

### 4.1 The Role of tsRNA in Neurodegenerative Diseases

#### 4.1.1 Abnormal tsRNA Shearing Causes Neuronal Abnormalities

CLP1 is an RNA kinase that is involved in tRNA maturation and is a member of the human tRNA splicing endonuclease (TSEN) complex [55]. CLP1 mutations can lead to abnormal tRNA shearing and produce tsRNA fragments that cause neuronal abnormalities [56, 57, 58, 59].

**FIGURE 5:**
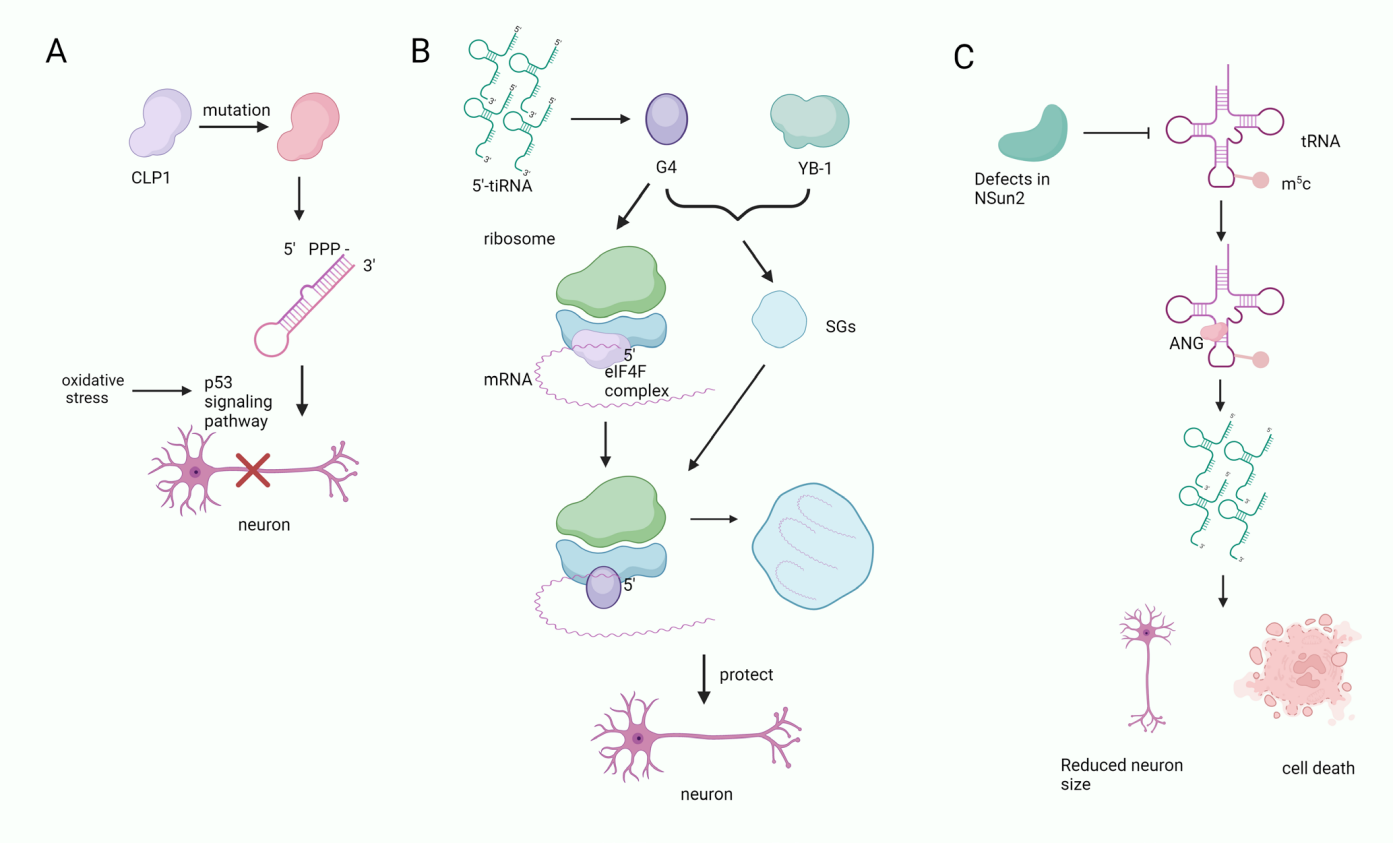
The role of tsRNA in neurodegenerative diseases. (A) Mutations in CLP1 cause aberrant tRNA exon junctions, leading to the accumulation of 5′ leading‐exon tRNA fragments. This makes the cells more susceptible to the effects of the p53 pathway, resulting in motor neuron loss. (B) tiRNAs form tetramers that displace the eIF4F complex. G4 and YB‐1 act together to induce the assembly of contingency granules (SGs). The SG protects neurons from cellular stress by recruiting translation‐stalled mRNAs to stop translation, thus protecting neurons from cellular stress. (C) Deficiency of NSun2 causes hypomethylation of tRNA; increased cleavage of tRNA by ANG causes accumulation of 5′ tiRNA; these lead to neuron size reduction, cell death, and make the cell more susceptible to stress.

Hanada et al. [56] found that mice with CLP1 kinase death progressively lose spinal motor neurons, leading to muscle denervation and paralysis. CLP1 kinase death results in aberrant exon junctions of tRNAs, resulting in 5′ bootstrap exonic tRNA fragments. These Tyr‐tRNA fragments make cells more susceptible to the p53 pathway, which is activated by oxidative stress and mediates motor neuron loss. This suggests that motor neuron damage caused by the death of CLP1 kinase may be due to the accumulation of Tyr‐tRNA fragments (Figure 5A). Furthermore, Schaffer et al. [59] showed that neurodegeneration in patients with mutations in the CLP1^R140H^ gene is caused by faulty tRNA splicing. The CLP1^R140H^ mutation leads to the accumulation of pre‐tRNA and depletion of mature tRNA in induced neurons. At the same time, they showed that Tyr tRNA 5′‐phospho‐3′‐exon fragment protects CLP1^R104H^ mutant cells from stress‐induced cell death. Compared to other tRNA fragments, the injection of 5′‐Tyr‐tRF into single‐cell zebrafish embryos causes more severe neural abnormalities [58]. Meanwhile, patients with severe motor sensory impairments, cortical hypoplasia, and microcephaly also carry a pure missense mutation in CLP1, which is also associated with tRNA splicing dysregulation [57]. The aforementioned studies demonstrated that abnormal tsRNA fragments resulting from CLP1 mutations can lead to neuronal abnormalities, subsequently contributing to the development of neurodegenerative conditions.

#### 4.1.2 Neuroprotective Effects of tsRNA

tiRNAs generated by ANG is able to exert a protective effect on neurons. tiRNA tetramer (G4) can displace the cap‐binding complex eIF4F from the capped mRNA and in return, inhibit translation initiation. Also, G4 can bind with the translation deterrent protein Y‐box binding protein 1 (YB‐1) and form the stress granules (SGs) [60]. SGs can recruit mRNAs that have stalled during translation as a way to terminate translation (Figure 5B) [61]. The G4 structure is essential for cytoprotective and pro‐survival functions. Its analog, 5′‐tiDNA^Ala^, spontaneously enters cells and protects motor neurons from the adverse effects of stress [62].

Endogenous angiopoietin knockout enhances stress‐induced motor neuron cell death in vitro [63]. It has been shown that tiRNA produced by ANG mediates stress‐induced RNA fragmentation in astrocytes to protect neurons [64]. Li et al. [65] showed that tiRNA prevented human neuron‐like cell apoptosis induced by mutations in granulin precursors. The above studies showed that the tiRNA generated by Argonaute has a neuroprotective effect.

#### 4.1.3 Hypomethylation Makes Neurons More Vulnerable to Stress

Defects in the methylation transferase NSun2 cause hypomethylation of tRNA, which in return increases angiopoietin‐mediated cleavage and leads to the accumulation of 5′ tRNA‐derived tsRNA and reduced protein translation. Accumulation of 5′ tRNA‐derived tsRNA and reduced protein translation leads to reduced cell volume, increased cell death; and activation of stress pathways in cortical, hippocampal, and striatal neurons (Figure 5C) [66]. Zhang et al. [67] found that the 30–40 nt portion of the cortex in AD patients had decreased levels of rsRNA‐5S, tsRNA‐Tyr, suggesting that this decrease in tsRNA‐Tyr may increase the susceptibility of neurons to oxidative stress. Wu et al. [68] found that tRF changed significantly in the hippocampus of AD patients. Additionally, it was shown that as NSun2 decreased, tRNA methylation decreased as well, causing tRF to accumulate. The above studies show that aberrant tRNA caused by aberrant NSun2 makes neuronal cells more susceptible to stress.

### 4.2 The Potential of tsRNAs as Therapeutic Targets for Neurodegenerative Diseases

#### 4.2.1 tsRNA as a Biomarker for the Diagnosis of Neurodegenerative Diseases

Magee, Londin, and Rigoutsos [69] have shown that tRFs are distributed differently in the prefrontal cortex, cerebrospinal fluid, and serum, with 58% of tRFs being unique to the prefrontal cortex; there are multiple differences in the abundance of tRFs in the brains of PD patients compared to normal subjects; they additionally examined the variations in tRFs between Parkinson's disease PDD and PND samples and found multiple tRFs expression differences between the two groups. Zhang et al. [70] compared the differences in tRFs in two models: the senescence‐accelerated mouse resistant 1 model as a control and the senescence‐accelerated mouse susceptible 8 model as a model for Parkinson's and Alzheimer's disease. Of the 570 tRF transcripts, 8 tRFs were differentially expressed. Grasbon‐Frodl et al. [71] analyzed 22 mitochondria‐encoded tRNAs from 20 Parkinson's disease patients by sequence analysis. They identified two new homozygous point mutations, one each in tRNA (Thr) (15950 G/A) and tRNA (Pro) (15965 T/C) genes. They demonstrated that these two new mutations may lead to the death of dopaminergic nerve cells in patients. As shown in the above study, tsRNAs can serve as PD biomarkers and can distinguish between PDD and PND. In zebrafish and mouse models of ALS, ELP3 overexpression affects the aggregation of susceptibility proteins and protects against ALS by oscillating tRNA modifications, suggesting a connection between tRNA modifications and motor neuron degeneration [72]. The 5′‐tRNA fragments TRV‐AAC4‐1.1 and TRA‐AGC6‐1.1 have been demonstrated to be considerably dysregulated in ALS models when compared to controls, suggesting that these two fragments may serve as possible biomarkers for ALS [73]. Hogg et al. [74] found that 5′‐t‐RNA‐Val‐CAC was highly expressed in the serum of an ALS mouse model, and 5′‐t‐RNA‐Val‐CAC was shown to be considerably higher in patients whose disease progressed slowly, suggesting that this tRNA has potential as a prognostic biomarker for ALS. In summary, tsRNA is a useful marker for the identification of neurodegenerative diseases.

#### 4.2.2 tsRNA Is a Promising Target for the Treatment of Neurodegenerative Diseases

Significant alterations in the expression of multiple tsRANs have been found following pharmacological treatment of neurodegenerative diseases and neurological recovery [75, 76, 77, 78, 79]. Bushen Tiansui formula (BSTSF), also known as Zhi Ling Tang, has been used for decades to treat Alzheimer's disease. BSTF‐treated animals showed a significant reversal of the expression levels of six tRNAs compared to untreated AD mice. These included tRNA‐Gln‐CTG‐002, tRNA‐Ser‐GCT‐001, tRNA‐Val‐AAC‐002, tRF‐Gly‐CCC‐011, tRF‐Thr‐CGT‐019, and tRF‐Ser‐GCT‐035. Learning and memory deficits were also ameliorated in AD mice [75]. Another herbal medicine, Buyang Huanwu decoction (BYHW), widely used for neurological recovery from stroke, has been shown to improve neurological deficits in both experimental studies and clinical trials. The expression of three tsRNAs was significantly reversed in cerebral hemorrhage rats treated with BYHWD. These three tsRNAs (rno‐tRFi‐Ser‐25a, rno‐tRF5‐Ala‐16a, and rno‐tRF5‐Glu‐29a) could exert therapeutic effects by regulating the FoxO signaling pathway, long‐term synaptic inhibition, and actin cytoskeleton organization of transforming growth factor β production [76, 77, 80]. The third herbal medicine, Xuefu Zhuyu decoction (XFZYD), has shown neuroprotective activity in brain injury treatment, such as alleviating neurological deficits [81], reducing neuroinflammation [82], and enhancing cognitive function [83]. In rats with traumatic brain injury treated with XFZYT, the expression of several tsRNAs was significantly reversed. These tsRNAs may ameliorate neurological damage and cognitive function by modulating the calcineurin signaling pathway, regulating developmental processes, and axon guidance [78, 79]. The above studies suggest that tsRNA could be a promising therapeutic target for neurological recovery.

### 4.3 tsRNA and Nucleotide Therapy

Oligonucleotide therapy is an emerging and promising therapeutic approach. It consists of double‐stranded small interfering RNAs (siRNAs), CRISPR‐Cas9 single guide RNAs, self‐replicating messenger RNAs (mRNAs), and single‐stranded antisense oligonucleotides [57, 84]. Among them, antisense oligonucleotides [57] is one of the most advanced RNA‐based therapeutic modalities. Multiple novel ASOs have successfully completed phase III clinical trials and received approval from the FDA [85]. tsRNAs have the potential to be utilized as therapeutic ASOs. This application may provide a new avenue for treating neurodegenerative diseases.

**FIGURE 6:**
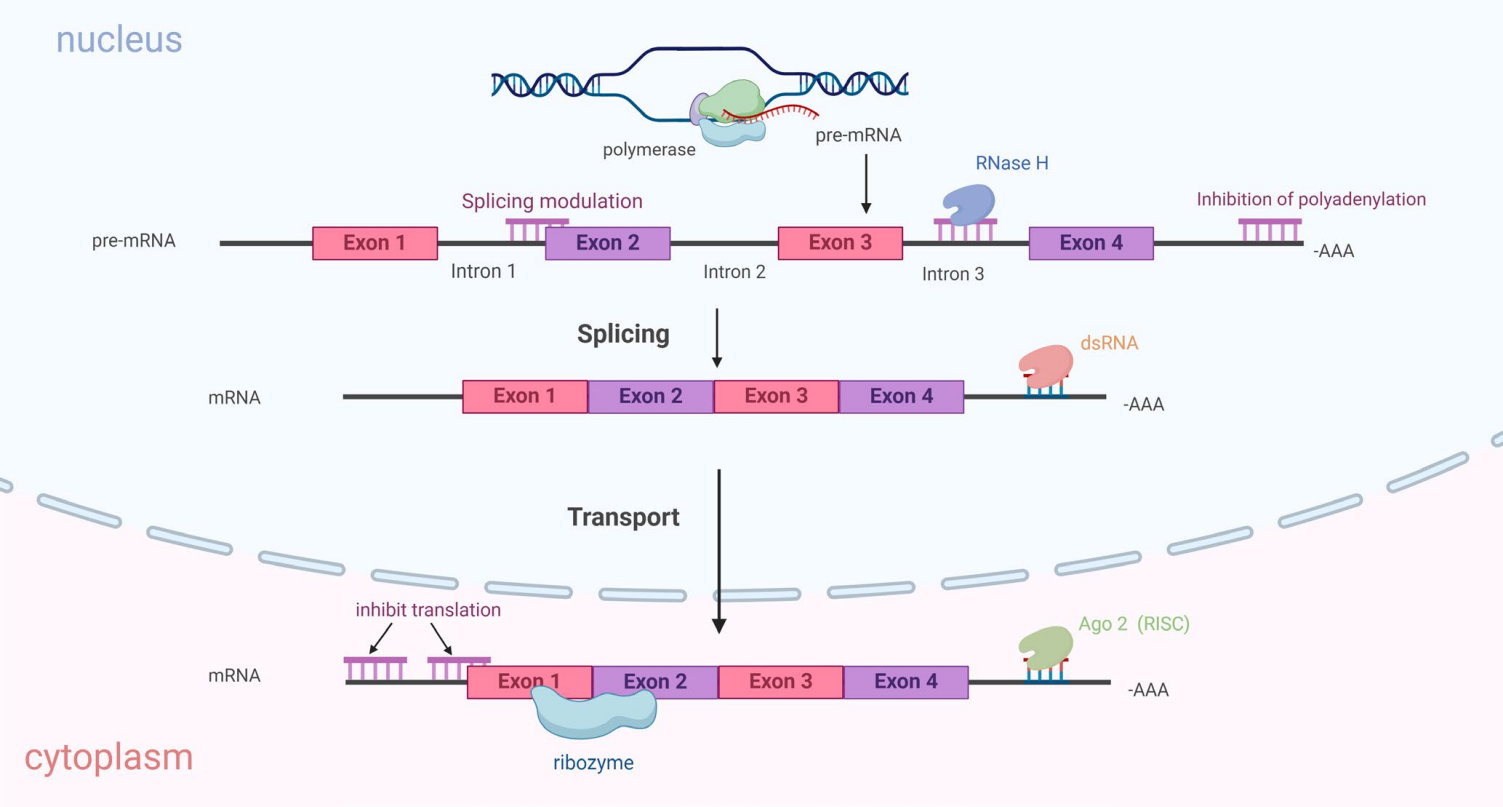
Mechanism of action of oligonucleotide drugs. At different steps during mRNA maturation, antisense oligonucleotides interact with each other and block mRNA function. ASO binds to pre‐mRNAs to regulate RNA splicing or inhibit polyadenylation, as well as to mRNAs for translational repression. Alternatively, ASO binds to pre‐mRNAs or mRNAs during mRNA genesis to degrade them by recruiting RNase H, AGO, ribozymes, and dsRNase.

ASO can bind to pre‐mRNA to recruit RNase H to degrade mRNA, regulate RNA shearing, or inhibit polyadenylation once RNA is transcribed from DNA into a precursor form (pre‐mRNA) [85, 86, 87]. It can also bind to mRNA in the cytoplasm for translational repression, recruit nuclease for RNA degradation, or form an inducible silencing complex with Ago2 for RNA repression (Figure 6).

Numerous studies have been conducted on ASO's potential for treating neurodegenerative diseases. There are currently five approved drugs for ASO in neurodegenerative diseases (Table 2). tsRNA is a prospective therapeutic target for the treatment of neurodegenerative diseases and plays an important role in neuroprotection and neuronal stress processes. tsRNA has been found to exhibit functions similar to miRNAs, binding to specific mRNAs through base pairing. This allows them to regulate mRNA translation or promote degradation [31]. Therefore, tsRNA could potentially target specific mRNAs through a similar mechanism, acting as a gene regulatory agent in ASO‐based therapies.

**TABLE 2:** Drugs approved for clinical use in the treatment of neurodegenerative diseases.

Drug	Target	Diseases	Mechanisms	Delivery method	References
Nusinersen	Survival motor neuron 2	SMA	Splicing modulation	Intrathecal	[88, 89]
Inotersen	Transthyretin	AD	RNase H	Subcutaneous	[90]
Patisiran	Transthyretin	AD	siRNA	Intravenous	[91]
Eteplirsen	Dystrophin	DMD	Splicing modulation	Intravenous	[92, 93]
Golodirsen	Dystrophin	DMD	Splicing modulation	Intravenous	[94]

## 5 Conclusions and Perspectives

tsRNAs have recently emerged as important regulatory molecules in neurodegenerative diseases. These small non‐coding RNAs are generated from tRNA fragments and play crucial roles in gene expression regulation and cellular processes. In this article, we discussed the biogenesis of tsRNA, its physiological and pathological functions in the central nervous system and neurological disorders, and its prospective use as a nucleic acid medication to treat NDDs. The pathogenesis of NDDs is complex and involves dysregulation of various molecular pathways. Emerging evidence suggests that tsRNAs play a critical role in the development and progression of neurodegenerative diseases. For example, certain tsRNAs have been found to target mRNAs encoding proteins involved in neuronal function and survival, leading to aberrant gene expression and neuronal dysfunction.

Overall, tsRNAs are emerging as important regulatory molecules in neurodegenerative diseases. Further research is needed to elucidate the specific roles of tsRNAs in the pathogenesis of these diseases and to explore their potential as therapeutic targets. Understanding the functions of tsRNAs in neurodegenerative diseases may provide new insights into disease mechanisms and lead to the development of novel treatment strategies.

## Author Contributions

Zhonghong Cao and Fengtang Yang have made contributions to the conception and design of the work. Tianqi Li and Hui Zhen have made contributions to the Literature collection and drafted the work. All the authors read and revised it.

## Ethics Statement

The authors have nothing to report.

## Consent

The authors have nothing to report.

## Conflicts of Interest

The authors declare no conflicts of interest.

## Data Availability

All data generated or analyzed during this study are included in this published article.
